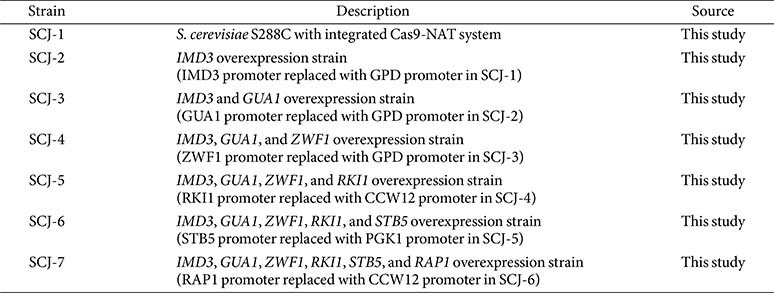# Scarless Genetic Engineering of *Saccharomyces cerevisiae* for Enhanced Guanosine Monophosphate Production as a Natural Flavor Enhancer

**DOI:** 10.4014/jmb.2508.08034

**Published:** 2025-12-09

**Authors:** Suk-Chae Jung, Hyunjoon Oh, Wonsik Eom, Yong-Su Jin, See-Hyoung Park, Kyungmoon Park, Hyun Gi Koh

**Affiliations:** 1 Department of Bioengineering, University of Illinois Urbana-Champaign, Urbana, Illinois 61801, USA; 2 DOE Center for Advanced Bioenergy and Bioproducts Innovation, University of Illinois at Urbana−Champaign, Urbana, Illinois 61801, USA; 3 Department of Food Science and Human Nutrition, University of Illinois at Urbana−Champaign, Urbana, Illinois 61801, USA; 4 Department of Fiber Convergence Material Engineering, Dankook University, Yongin-si 16890, Republic of Korea; 5 Department of Biological and Chemical Engineering, Hongik University, Sejong 30016, Republic of Korea

**Keywords:** *S. cerevisiae*, GMP, guanosine 5'-monophosphate, umami, savory

## Abstract

*Saccharomyces cerevisiae* and *Cyberlindnera jadinii* are widely utilized in the natural food seasoning industry as sources of flavor enhancing nucleotides such as inosine monophosphate (IMP) and guanosine monophosphate (GMP), which contribute to umami taste and support sodium reduction in food. However, wild type yeast strains produce GMP at levels that are inadequate for industrial scale applications, necessitating metabolic engineering strategies to increase production efficiency. This study employed a CRISPR-Cas9-based scarless genome engineering approach to enhance GMP biosynthesis in *S. cerevisiae* via promoter replacement. The key genes *IMD3* and *GUA1*, responsible for converting IMP to GMP, were overexpressed to redirect purine flux toward GMP production. To address precursor limitations, *ZWF1* and *RKI1*, involved in the pentose phosphate pathway, were also overexpressed. In parallel, the expression of *STB5* and *RAP1* was increased to enhance NADPH regeneration and relieve transcriptional bottlenecks. As a result, the final engineered strain SCJ-7 demonstrated a 1.77-fold increase in GMP titer and a 1.40-fold increase in GMP content during flask fermentation compared to the wild-type. In fed-batch fermentation, GMP titer was further improved by 27.6%. These findings demonstrate that combining metabolic flux enhancement with transcriptional regulation provides an effective and scalable strategy for boosting GMP production in *S. cerevisiae*, offering strong potential for industrial application in the food industry.

## Introduction

The growing demand in the food industry has led to the widespread use of chemically synthesized seasonings, such as monosodium glutamate (MSG) and hydrolyzed vegetable protein (HVP) [1, 2]. However, increasing consumer concerns about synthetic additives and a growing preference for natural ingredients have simultaneously driven interest in natural flavor enhancers. Among them, yeast extract has emerged as a promising alternative due to its ability to deliver rich umami taste through naturally occurring compounds [3, 4]. Yeast cells are composed of more than 50% protein, including amino acids and peptides, and are therefore considered a valuable source of flavor-enhancing substances such as glutamic acid, inosine monophosphate (IMP), and guanosine monophosphate (GMP) [5, 6]. Despite this potential, the natural production levels of IMP and GMP in wild-type yeast strains remain insufficient for industrial-scale applications, creating a strong need for engineered strains with enhanced purine nucleotide biosynthetic capacity.

IMP and GMP are purine nucleotides that serve as key flavor enhancers, especially in the development of the umami taste, recognized as the fifth basic taste alongside sweet, sour, salty, and bitter. MSG is one of the most well-known umami compounds [7-9]. When mixed with glutamic acid (GA) at a 1:1 ratio, IMP can enhance the umami flavor by approximately sevenfold, while GMP can boost it up to thirtyfold. These nucleotides also contribute to sodium reduction in food products, aligning with current health-conscious consumption trends [10-14].

*Saccharomyces cerevisiae* and *Cyberlindnera jadinii*, both generally recognized as safe (GRAS) organisms, have been widely used as host strains for producing purine nucleotides [15-17]. Traditionally, random mutagenesis has been applied to improve their productivity; however, this approach suffers from key limitations, such as the inability to trace specific genomic changes and the potential for spontaneous reversion. To overcome these drawbacks, a more rational and stable method involving genetic engineering is required. By selectively overexpressing key genes, purine nucleotide production can be significantly enhanced. Furthermore, genomic integration ensures long-term genetic stability and consistent metabolite yields, which are critical for industrial applications. This shift from random mutagenesis to precise genome engineering marks a significant advancement in microbial strain development for natural food ingredients.

**Fig. 1:**
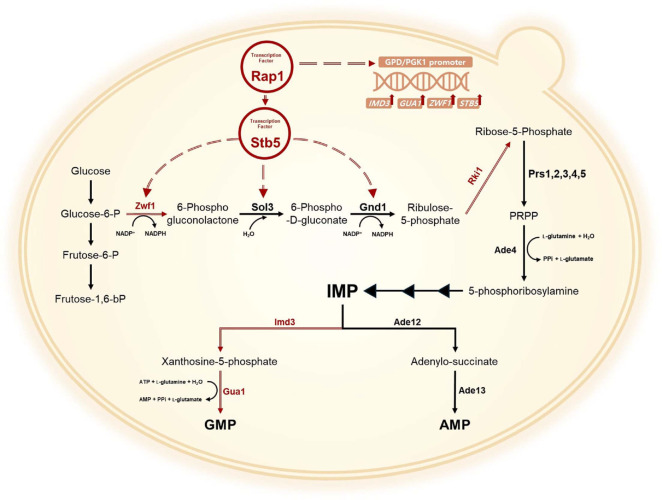
A scheme of the GMP biosynthesis pathway in *S. cerevisiae*. Enzyme abbreviations are as follows: Zwf1, cytoplasmic glucose-6-phosphate dehydrogenase; Sol3, 6-phosphogluconolactonase; Gnd1, 6-phosphogluconate dehydrogenase; Stb5, Salt tolerance breakpoint 5 (Transcription factor); Prs1,2,3,4,5, 5-phospho-ribosyl-1(alpha)-pyrophosphate synthetase; Ade4, Phosphoribosylpyrophosphate amidotransferase; Imd3, Inosine monophosphate dehydrogenase; Gua1, Guanosine monophosphate synthase; Ade12, Adenylosuccinate synthase; Ade13, Adenylosuccinate lyase.

In *S. cerevisiae*, the biosynthesis of IMP and GMP involves glycolysis, the pentose phosphate (PP) pathway, and the purine biosynthetic pathway [18, 19]. Ribulose 5-phosphate generated through the pentose phosphate pathway is converted to ribose 5-phosphate by *RKI1*, which subsequently enters the purine biosynthesis pathway to form IMP. This intermediate can follow two distinct metabolic routes: conversion into AMP via *ADE12* and *ADE13*, or conversion into GMP via *IMD3* and *GUA1* (Fig. 1). Previous studies have shown that nucleotide production can be improved through metabolic pathway engineering. For example, in *Ashbya gossypii*, overproduction of riboflavin was achieved by mutating *AgADE4*, which encodes PRPP amidotransferase, to relieve feedback inhibition [20]. The same research group also enhanced inosine and guanosine production by overexpressing *ADE4* while simultaneously knocking out *ADE12* and *PNP1* [21].

Building on these insights, this study employed a scarless CRISPR-Cas9-based approach to metabolically engineer *S. cerevisiae* for enhanced purine nucleotide production [22, 23]. This strategy eliminates residual genetic scars from marker-assisted integration and relies solely on homologous regulatory elements, ensuring both genetic stability and regulatory safety for food-related applications. Strong homologous promoters were used to replace the native promoters of *ZWF1*, *RKI1*, *IMD3*, and *GUA1*, thereby increasing pathway flux toward purine nucleotide biosynthesis. In addition, transcription factors *RAP1* and *STB5* were overexpressed to further elevate global transcriptional capacity. The performance of the engineered strain was evaluated through both flask-scale and fed-batch fermentation, providing insight into its industrial applicability.

## Materials and Methods

### Strains and Media

*Escherichia coli* DH5α (Invitrogen, USA) was used for plasmid construction and propagation. *E. coli* was grown in the LB Lenox medium (5 g/l yeast extract, 10 g/l tryptone, 5 g/l NaCl, pH 7.0) supplemented with Ampicillin (100 μg/ml) at 37°C. *S. cerevisiae* S288C strain was grown in YPD medium (10 g/l yeast extract, 20 g/l peptone and 20 g/l glucose) at 30°C. Yeast strains transformed with plasmids containing antibiotic markers were propagated on YPD plates supplemented with Nourseothricin (clonNAT) (100 μg/ml) and/or geneticin G418 (200 μg/ml). For GMP production, defined media were used for both flask and bioreactor fermentations. The defined medium consisted of 30 g/l D-glucose, 0.5 g/l MgSO_4_∙7H_2_O, 10 g/l NH_4_H_2_PO_4_, 0.6 g/l KH_2_PO_4_, 1 ml/l of trace metal solution, and 10 ml/l of vitamin solution. Trace metal solution contained 7 g/l ZnSO_4_∙7H_2_O, 12 g/l FeSO_4_∙7H_2_O, 0.2 g/l CuSO_4_, 4 g/l MnSO_4_∙4H_2_O. The vitamin solution contained 1.064 g/l thiamine HCl, 0.532 g/l pyridoxine HCl, and 0.532 g/l pantothenate.

### Comparison of mRNA Expression Level

Quantitative PCR (qPCR) was performed to compare the mRNA expression levels of target genes between the control and engineered strains. Both strains were cultured in a shaking incubator at 30°C and 200 rpm for 36 h. Total RNA was extracted using the YeaStar RNA Kit (Zymo Research, USA), and 1 μg of RNA was reverse transcribed into cDNA using the GoScript Reverse Transcription System (Promega, USA) in a 20 μl reaction volume. The resulting cDNA was diluted to a concentration of 50 ng/μl for subsequent analysis. qPCR was carried out in duplicate using the CFX Connect Real-Time PCR Detection System (Bio-Rad, USA). The *S. cerevisiae* actin gene (*ACT1*) was used as the internal reference. Primer sequences for *ACT1* and target genes are listed in Table S4. Primer titration and melt-curve (dissociation) analyses were conducted to ensure the absence of primer dimers and nonspecific amplification. Cycle threshold (C_t_) values for both the reference and target genes were obtained using automatic baseline setting and a manually adjusted threshold.

### Plasmids and Strain Construction

The strains used in this study are summarized in Table 1. Plasmids, primers, and guide RNA target sequences are listed in Tables S1, S2, and S3, respectively. Recombinant DNA constructs for guide RNA expression were generated using the FAST cloning method [24]. Yeast transformation was carried out using the lithium acetate method with single-stranded carrier DNA and polyethylene glycol (PEG) [25]. Cas9-NAT, gRNA expression vectors, and donor DNA fragments were co-transformed into yeast strains [2, 26]. Putative transformants on selection plates were confirmed by colony PCR with 20mM NaOH solution [27-29]. Detailed procedures for plasmid construction and strain generation are provided in the Supplementary Materials. To remove the gRNA expression vector after completing single-gene editing, the engineered strain was streaked on YPD plates containing clonNAT and incubated at 30°C for 72 h. Individual colonies were subsequently transferred onto YPD plates supplemented with clonNAT and G418 to confirm the loss of the gRNA plasmid. Finally, to eliminate the CRISPR–Cas9 expression vector and obtain a marker-free, scarless strain, colonies were streaked on standard YPD plates and screened for loss of clonNAT resistance.

### Fermentation Experiments

For the flask fermentation, engineered yeast strains were retrieved from frozen stocks and pre-cultured in 5 ml of YPD medium at 30°C and 250 rpm for 1~2 days. The precultured yeast cells were then inoculated into 100 ml of defined medium in a 500 ml baffled flask and incubated at 30°C and 200 rpm.

For Fed-batch fermentation, engineered yeast strains were streaked on agar plates from frozen stocks and precultured in 5 ml of YPD medium at 30°C and 250rpm for 24 h. A 1 ml aliquot of the culture was transferred to 100 ml of YPD in a 500 ml baffled flask for seed culture and incubated under the same conditions for another 24 h. Subsequently, 100 ml of the seed culture was inoculated into 1.5 l of YPD medium in a 5-l bioreactor. Batch fermentation was conducted at 30°C with an airflow rate of 1 vvm and agitation at 300 rpm. At 16 h after the initiation of fermentation, the culture was supplemented with 50 ml of a salt solution containing MgSO_4_·7H_2_O, NH_4_H_2_PO_4_, and KH_2_PO_4_. Additionally, trace metal and vitamin solutions were added at concentrations of 1 ml/l and 10 ml/l, respectively. A concentrated glucose solution (300 g/l) was continuously fed at a rate of 1ml/min for 12 h. The pH was controlled at 5.5 using 8% NH_4_OH.

### GMP Determination

HPLC sample preparation was performed as follows. 5 ml of yeast culture was centrifuged at 4,000 rpm for 10 min. The supernatant was discarded, and the cell pellets were washed three times with 30 ml of distilled water. Guanine standard solutions were prepared at concentrations of 5, 10, 20, 30, and 40 mg. To each sample and standard, 45 ml of 0.5 M perchloric acid (HClO_4_) was added and incubated at 37°C for 90 min. Subsequently, 0.9 ml of 60% HClO_4_ was added, and the mixture was heated at 100°C for 2 h to hydrolyze nucleotides into their corresponding bases. Under these conditions, GMP was degraded to guanine, which was then quantified by HPLC to calculate the total GMP content [30]. The resulting solution was neutralized with sodium bicarbonate (NaHCO_3_) until the pH indicator (litmus paper) turned green. The solution was then filtered through a 0.2 μm PVDF membrane filter and subjected to HPLC analysis. Intracellular GMP analysis was conducted using a Thermo Ultimate 3000 HPLC system equipped with a CAPCELL PAK C18 UG 120Å reversed-phase column (4.6 mm × 150 mm, 5 μm; Osaka Soda, Japan). GMP was quantified by monitoring UV absorbance at 254 nm. Isocratic elution was performed using 0.05 M potassium phosphate buffer (pH 6.3) as the mobile phase, with a flow rate of 1.0 ml/min over a 20 min run.

### Statistical Analysis

All experiments were performed in biological triplicates (*n* = 3), and the results are presented as mean ± standard error (SE). Statistical analyses were conducted using Student’s *t*-test to evaluate differences between the control and engineered strains. A *p*-value of less than 0.05 was considered statistically significant. Significance levels are indicated in the figures as *p < 0.05, **p < 0.01, and ***p < 0.001. All statistical calculations were performed using Microsoft Excel.

## Results and Discussion

### Enhancement of Metabolic Flux toward GMP Biosynthesis by Overexpression of *IMD3* and *GUA1*

In the purine biosynthetic pathway, IMP is a central branch point that can be converted into both GMP and AMP, catalyzed by *IMD3* and *GUA1*, or *ADE12* and *ADE13*, respectively [18] (Fig. 1). Given that AMP synthesis competes with GMP formation, a significant portion of IMP is diverted toward AMP, limiting GMP accumulation. To enhance GMP biosynthesis and increase the total guanylate pool in *S. cerevisiae*, the metabolic flux toward GMP was increased by overexpressing *IMD3* and *GUA1*.

**Fig. 2:**
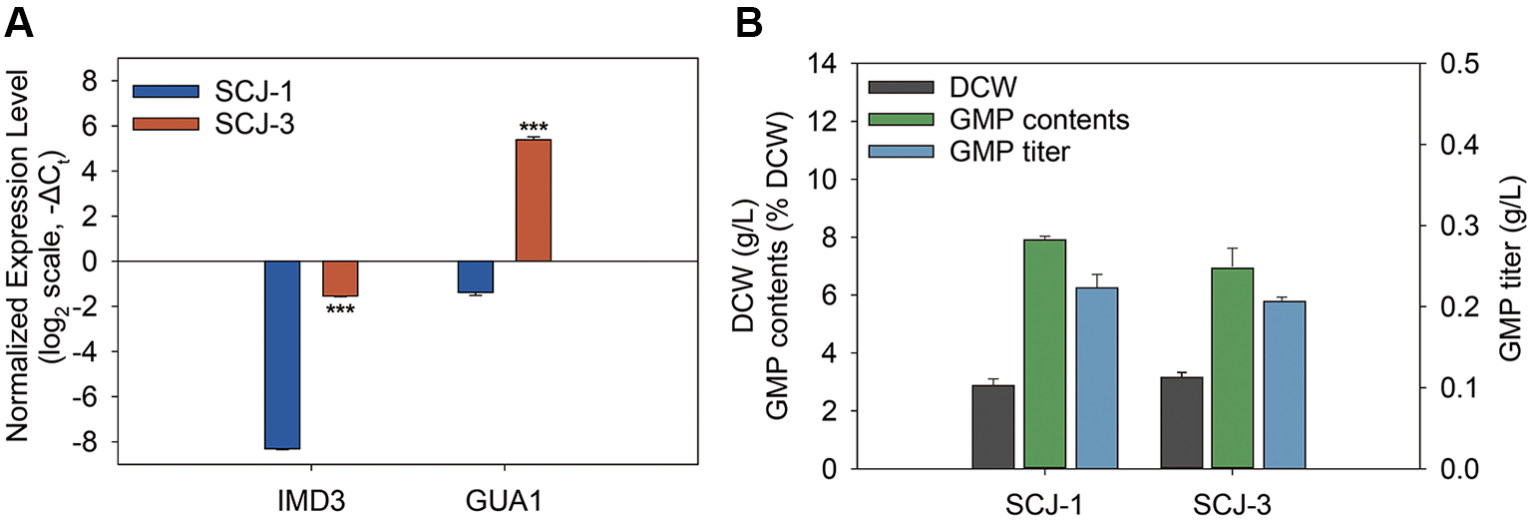
Comparison of mRNA expression, biomass, and GMP production between wild-type (SCJ-1) and *IMD3*/*GUA1*-overexpressing strain (SCJ-3). (**A**) Relative expression levels of *IMD3* and *GUA1* in SCJ-1 and SCJ-3, normalized to *ACT1*, and shown as –ΔC_t_ values on a log_2_ scale. Negative values indicate lower expression compared to *ACT1* in each strain. (**B**) Comparison of dry cell weight (DCW), GMP content (% DCW), and GMP titer (g/l) between SCJ-1 and SCJ-3 on day 3 of flask fermentation. Data are presented as mean ± standard error (*n* = 3). Statistical significance relative to SCJ-1 was determined using Student’s *t*-test (*p < 0.05, **p < 0.01, ***p < 0.001).

Overexpression of the targeted genes was accompanied by the replacement of their native promoters with strong, well-characterized promoters via homologous recombination, facilitated by CRISPR-Cas9. To implement this strategy, strain SCJ-2 was constructed by replacing the native *IMD3* promoter with the GPD promoter, which is known for its robust activity in *S. cerevisiae* [31].The homologous GPD promoter fragment was amplified using primers JSC-Pri-18 and JSC-Pri-59, then co-transformed with the gRNA plasmid JSC-P-5 into strain SCJ-1. Following this, strain SCJ-3 was generated by replacing the native *GUA1* promoter with the same GPD promoter. For this step, the GPD promoter fragment was amplified using primers JSC-Pri-20 and JSC-Pri-21 and co-transformed with the gRNA plasmid JSC-P-7 into strain SCJ-2. To confirm the effectiveness of this approach, qPCR analysis was performed, showing that *IMD3* and *GUA1* transcript levels in SCJ-3 were increased by 108-fold and 109-fold, respectively, compared to those in the parental strain SCJ-1 (Fig. 2A). The difference in fold increases can be attributed to the initial expression levels of *IMD3* and *GUA1* in the parental strain. While *IMD3* had a higher baseline expression level, leading to a 108-fold increase, *GUA1* had a much lower initial expression, resulting in a more substantial 109-fold increase following the promoter replacement. These results highlight the effectiveness of the promoter exchange strategy, with the degree of gene expression enhancement varying according to the initial levels in the parental strain.

However, despite the enhanced flux from IMP to GMP, no significant changes in dry cell weight (DCW) (2.9 g/l vs. 3.1 g/l), GMP content (0.23% vs. 0.22%), or GMP titer (0.22 g/l vs. 0.21 g/l) were observed between SCJ-1 and SCJ-3 (Fig. 2B). These results suggest that while the metabolic flux from IMP to GMP was successfully enhanced, upstream bottlenecks, such as limited availability of ribose-5-phosphate or phosphoribosyl pyrophosphate (PRPP), may have constrained overall purine biosynthesis capacity, leading to no significant increase in GMP production [32]. Furthermore, Gua1 activity requires glutamine and ATP as cofactors, and insufficient levels may impair enzyme function even under high expression conditions [33]. Additionally, the accumulation of excessive GMP might have caused feedback inhibition, disrupting nucleotide balance and impairing GMP synthesis [34, 35].

### Increasing Flux through the Pentose Phosphate and Nucleotide Synthesis Pathways

The enhancement of flux from IMP to GMP alone did not sufficiently increase GMP production, suggesting that upstream precursors may be the limiting factor. To address this, metabolic flux was redirected toward the pentose phosphate (PP) and nucleotide synthesis pathways by overexpressing *ZWF1* and *RKI1*, two key genes involved in these processes. *ZWF1* encodes glucose-6-phosphate dehydrogenase, a rate-limiting enzyme in the oxidative PP pathway, while *RKI1* encodes ribose-5-phosphate isomerase, which catalyzes the interconversion of ribulose-5-phosphate and ribose-5-phosphate, a direct precursor for purine nucleotide biosynthesis.

**Fig. 3:**
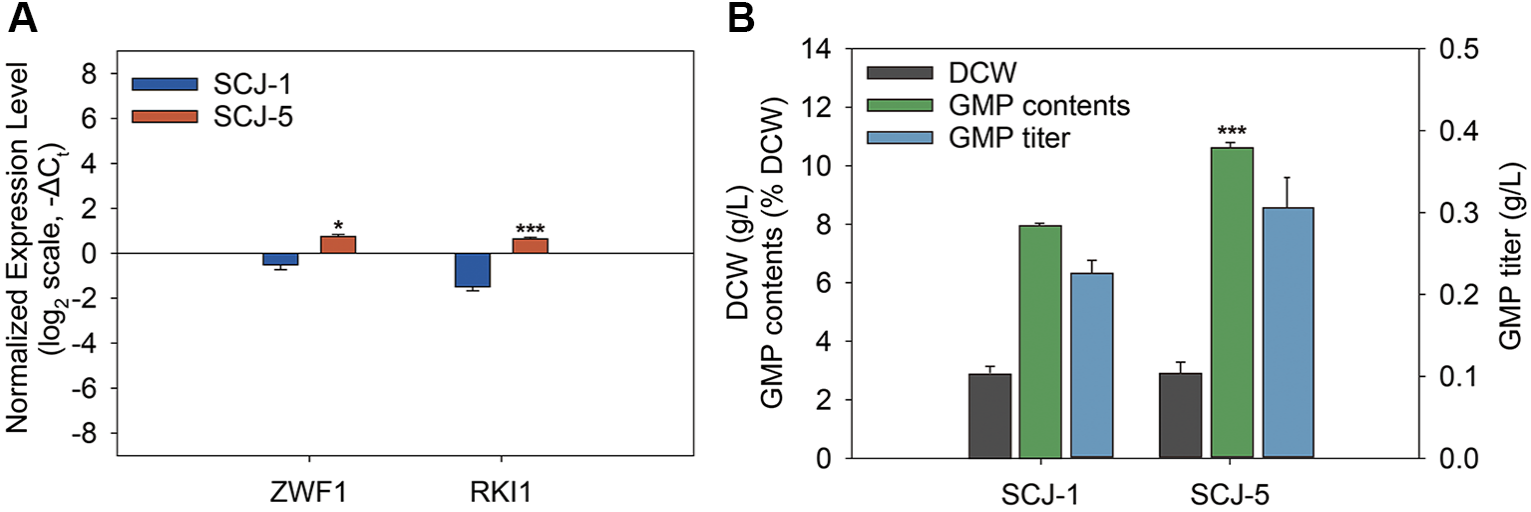
Effect of *ZWF1* and *RKI1* overexpression on GMP titer. (**A**) Relative expression levels of *ZWF1* and *RKI1* in SCJ-1 and SCJ-5, normalized to *ACT1*, and shown as –ΔC_t_ values on a log_2_ scale. Negative values indicate lower expression compared to *ACT1* in each strain. (**B**) Comparison of dry cell weight (DCW), GMP content (% DCW), and GMP titer (g/l) between SCJ-1 and SCJ-5 on day 3 of flask fermentation. Values represent the mean ± standard error (*n* = 3). Statistical significance relative to SCJ-1 was determined using Student’s *t*-test (*p < 0.05, **p < 0.01, ***p < 0.001).

To enhance the expression of these genes, promoter replacement was performed, using the GPD promoter for *ZWF1* and the CCW12 promoter for *RKI1*. The GPD promoter fragment was amplified with primers JSC-Pri-22 and JSC-Pri-23 and used to replace the native ZWF1 promoter in strain SCJ-3, generating strain SCJ-4. Similarly, the CCW12 promoter was introduced into *RKI1* in SCJ-4 using primers JSC-Pri-24 and JSC-Pri-25, resulting in strain SCJ-5. The CCW12 promoter is a strong constitutive promoter, commonly used in yeast for stable and high-level gene expression [36]. It is particularly effective because it remains active even under glucose-limited conditions and is not regulated by *RAP1*, allowing for sustained expression of target genes in engineered strains [37]. qPCR analysis confirmed that *ZWF1* and *RKI1* transcript levels in SCJ-5 increased by 2.4-fold and 4.3-fold, respectively, compared to the parental strain SCJ-1 (Fig. 3A). Although DCW remained largely unchanged between SCJ-1 (3.1 g/l) and SCJ-5 (3.0 g/l), GMP content in SCJ-5 increased from 7.7% to 10.2% of DCW, representing a 32% improvement. Consequently, GMP titer rose from 0.22 g/l in SCJ-1 to 0.30 g/l in SCJ-5, corresponding to a 36% increase (Fig. 3B). These results demonstrate that the overexpression of *ZWF1* and *RKI1* represents an effective strategy for enhancing total GMP content, primarily by improving precursor and cofactor availability. In particular, *ZWF1* contributes to NADPH generation, which supports reductive biosynthetic processes, while *RKI1* promotes the formation of ribose-5-phosphate, a critical sugar moiety that serves as the backbone of purine nucleotides.

### Enhancing Transcriptional Regulation to Promote GMP Biosynthesis

To further improve GMP production, transcriptional regulation was targeted in addition to pathway-specific enzyme overexpression. Based on the effectiveness of PPP enhancement through *ZWF1* and *RKI1* overexpression, transcription factors regulating this pathway were selected as new engineering targets. One such factor is *STB5*, which is known to activate multiple genes in the oxidative branch of the PPP, including *ZWF1*, *SOL3*, *TKL1*, *GND1*, *GND2*, *RKI1*, and *TAL1* [38]. In addition, *STB5* supports NADPH regeneration by regulating *GOR1*, *IDP2*, *YEF1*, and *ALD6*, and contributes to oxidative stress resistance [39]. To enhance *STB5* expression, its native promoter was replaced with the strong constitutive *PGK1* promoter in strain SCJ-5, resulting in the construction of strain SCJ-6. The *PGK1* promoter is a well characterized glycolytic promoter widely used for stable and high level expression in *S. cerevisiae*, and it operates independently of *STB5* mediated regulation [40]. Namely, strain SCJ-6 harbors five key genes (*IMD3*, *GUA1*, *ZWF1*, *RKI1*, and *STB5*) overexpressed under the control of strong constitutive promoters compared to the parental strain SCJ-1. However, many of the promoters used in this strain, including *GPD* and *PGK1*, are known to depend on *RAP1* for transcriptional activation [41, 42]. In particular, the GPD promoter used for *IMD3*, *GUA1*, and *ZWF1* shares regulatory elements with several glycolytic gene promoters that are activated by *RAP1* [42-44].This increased reliance on *RAP1* may have saturated its availability, limiting transcriptional efficiency and constraining GMP production. To alleviate this potential limitation, *RAP1* expression was enhanced in strain SCJ-6 by replacing its native promoter with the constitutive CCW12 promoter, resulting in strain SCJ-7. The CCW12 promoter is not regulated by *RAP1* and maintains strong transcriptional activity even under glucose-limited conditions [37, 41], making it suitable for ensuring stable *RAP1* expression without introducing regulatory feedback.

**Fig. 4:**
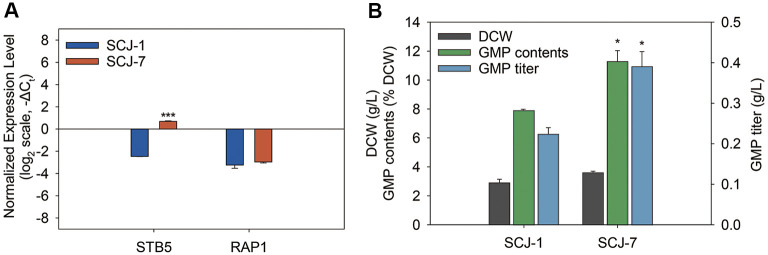
Comparison of normalized mRNA expression, biomass, and GMP production between wild-type (SCJ-1) and *STB5*/*RAP1*-engineered strain (SCJ-7). (**A**) Relative expression levels of *STB5* and *RAP1* in SCJ-1 and SCJ-7, normalized to *ACT1*, and shown as –ΔC_t_ values on a log_2_ scale. Negative values indicate lower expression compared to *ACT1* in each strain. (**B**) Comparison of dry cell weight (DCW), GMP content (% DCW), and GMP titer (g/l) between SCJ-1 and SCJ-7 on day 3 of flask fermentation. Data are presented as mean ± standard error (*n* = 3). Statistical significance relative to SCJ-1 was determined using Student’s *t*-test (*p < 0.05, **p < 0.01, ***p < 0.001).

qRT-PCR analysis of SCJ-7 revealed an 8.9-fold increase in *STB5* transcript levels and a 1.2-fold increase in *RAP1* compared to the parental strain SCJ-1 (Fig. 4A). This confirmed that both transcription factors were successfully overexpressed in the final engineered strain. When SCJ-7 was cultivated under flask fermentation conditions, notable improvements in cell growth and GMP production were observed. Specifically, dry cell weight (DCW) increased from 2.9 g/l to 3.6 g/l, GMP content rose from 7.7% to 10.8% of DCW, and GMP titer increased from 0.22 g/l to 0.39 g/l, representing 24%, 40%, and 77% improvements, respectively, compared to SCJ-1 (Fig. 4B). These results suggest that overexpression of *RAP1* alleviated transcriptional bottlenecks likely caused by excessive reliance on *RAP1*-dependent promoters, thereby enhancing the expression efficiency of engineered pathways. In parallel, elevated *STB5* expression appears to have further boosted flux through the pentose phosphate pathway, improving NADPH regeneration and redox homeostasis. This enhancement in oxidative stress tolerance may have contributed to greater cellular robustness, thereby supporting higher biomass accumulation during cultivation and facilitating increased GMP biosynthesis [38, 45]. The observed increase in biomass may also reflect improved metabolic capacity, supported by higher NADPH availability and more efficient precursor utilization. Taken together, these enhancements reflect the cumulative effect of transcriptional reinforcement and pathway-level optimization accumulated across engineering steps. The final strain SCJ-7 exhibited the highest GMP productivity among all variants, demonstrating the value of fine-tuning both gene expression and metabolic capacity for strain improvement.

### Fed-Batch Fermentation

**Fig. 5:**
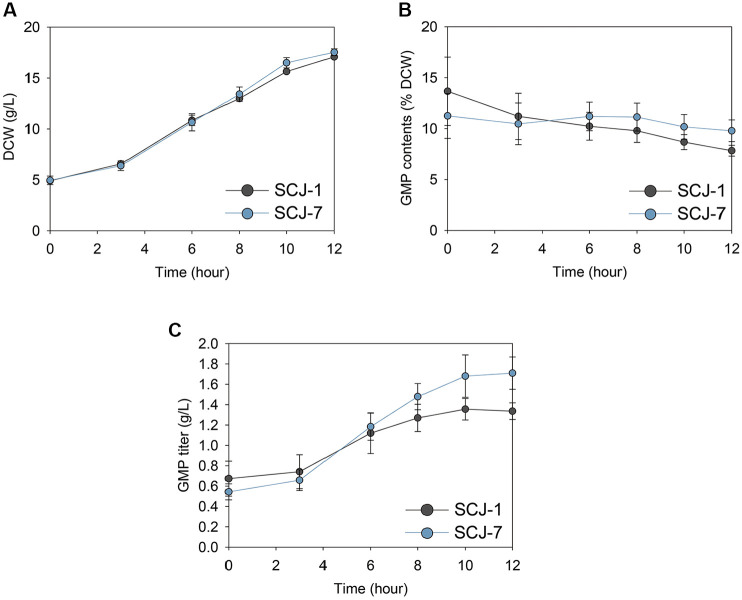
Bioreactor fermentation performance of SCJ-1 and SCJ-7. (**A**) Comparison of dry cell weight (DCW) during fed-batch fermentation. (**B**) GMP content (% DCW) and (**C**) total GMP titer (g/l) measured after 16 hours of fermentation. Values represent the mean ± standard error (*n* = 3).

To assess whether the GMP -overproducing phenotype of SCJ-7 could be maintained at larger scales, fed-batch fermentation of SCJ-1 and SCJ-7 was conducted in a 5 L bioreactor using a two-phase strategy (Fig. 5). The first phase aimed to maximize biomass accumulation in YP medium containing 30 g/l glucose, followed by a production phase that incorporated trace metal and vitamin supplementation along with continuous feeding of a 300 g/l glucose solution at 1.0 ml/min, as described in the materials and methods section. During the production phase, both strains exhibited comparable biomass accumulation, with SCJ-7 showing a slightly higher dry cell weight (DCW) on day 12 (Fig. 5A). In contrast, GMP content was significantly higher in SCJ-7, reaching 9.79% of DCW compared to 7.83% in SCJ-1, corresponding to a 25% increase (Fig. 5B). Consistently, GMP titer in SCJ-7 reached 1.71 g/l, representing a 27.6% improvement over the 1.34 g/l observed in SCJ-1 (Fig. 5C). These results demonstrate that SCJ-7 sustained enhanced GMP production even under high-cell-density conditions, supporting its potential applicability to industrial-scale processes.

To date, several studies have attempted to increase RNA or nucleotide content in *S. cerevisiae* and other yeasts through genetic engineering for seasoning applications. However, most of these reports only presented the percentage increase in total RNA content relative to the parental strain, without providing quantitative values for nucleotide production [46-50]. One of the few studies that reported actual titers demonstrated the secretion of combined IMP and GMP up to 0.4 g/l in *Ashbya gossypii* through pathway engineering [21]. Although this result cannot be directly compared due to differences in species and secretion-based production, it provides a useful reference point.

Therefore, this study holds particular significance as it quantitatively demonstrates intracellular GMP accumulation through stepwise promoter replacement and regulatory gene engineering in *S. cerevisiae*, achieving a GMP titer of 1.71 g/l in a food-grade host. The improvements observed in both yield titer and cellular GMP content highlight the robustness of the engineered regulatory and metabolic framework, even under nutrient-limited, large-scale fermentation conditions.

## Conclusion

This study presents a rational engineering strategy to enhance GMP production in *S. cerevisiae* by systematically improving both metabolic flux and transcriptional regulation, as demonstrated by a series of engineered strains (Table 1). The purine biosynthetic pathway was redirected toward GMP by overexpressing *IMD3* and *GUA1*, which alone led to limited improvements. To address upstream constraints, *ZWF1* and *RKI1* were overexpressed to boost precursor and cofactor availability via the pentose phosphate pathway, resulting in a 1.36-fold increase in GMP titer. Further enhancement was achieved by reinforcing transcriptional control through the overexpression of *STB5* and *RAP1*, which alleviated potential bottlenecks associated with the saturation of *RAP1*-dependent promoters. The final engineered strain, SCJ-7, demonstrated robust GMP overproduction in both flask and bioreactor fermentations, with a 1.77-fold increase in GMP titer (from 0.22 g/l to 0.39 g/l) under flask conditions and a 27.6% improvement under fed-batch conditions (1.71 g/l).

These findings demonstrate that combining pathway-specific metabolic engineering with targeted transcriptional enhancement offers a robust and scalable platform for GMP overproduction. The multi layered approach developed in this study provides a reliable alternative to traditional mutagenesis-based methods and lays a strong foundation for future strain optimization. Further integration of cofactor balancing and dynamic regulation may unlock even higher productivity, contributing to the industrial production of GMP based ingredients for the food and biotechnology sectors.

## Supplemental Materials

Supplementary data for this paper are available on-line only at http://jmb.or.kr.



## Figures and Tables

**Table 1:** Strain used or constructed in this study.