# Supplementary material for: Scarless Genetic Engineering of Saccharomyces cerevisiae for Enhanced Guanosine Monophosphate Production as a Natural Flavor Enhancer
Source: J Microbiol Biotechnol. 2025 Dec 9;35:e2508034. doi: 10.4014/jmb.2508.08034 (PMC12706149; doi:10.4014/jmb.2508.08034)
Supplement: Supplementary file 1 [file jmb-35-e2508034-supple.pdf]

J. Microbiol. Biotechnol. 2025. 35: e2508034

https://doi.org/10.4014/jmb.2508.08034

Supplementary Tables

Scarless Genetic Engineering of *Saccharomyces cerevisiae* for Enhanced Guanosine Monophosphate Production as a Natural Flavor Enhancer

Suk-Chae Jung[1], Hyunjoon Oh[2,3], Wonsik Eom[4], Yong-Su Jin[2,3], See-Hyoung Park[5], Kyungmoon Park[5], and Hyun Gi Koh[5*]

[1]Department of Bioengineering, University of Illinois Urbana-Champaign, Urbana, IL, USA

[2]DOE Center for Advanced Bioenergy and Bioproducts Innovation, University of Illinois at Urbana−Champaign, Urbana, Illinois 61801, USA

[3]Department of Food Science and Human Nutrition, University of Illinois at Urbana−Champaign, Urbana, Illinois 61801, USA

[4]Department of Fiber Convergence Material Engineering, Dankook University, Yongin-si, Republic of Korea

[5]Department of Biological and Chemical Engineering, Hongik University, Sejong 30016, Republic of Korea

Corresponding author: Hyun Gi Koh , hgkoh@hongik.ac.kr

Table S1. Primer sequences for construction of gRNA expression plasmids. The upper case of gRNA-Pri-2, 3, 4, 5, 6, and 7 were presented with target sequences.

Target gene	Primer name	Primer sequence	gRNA expression vector
IMD3	gRNA-Pri-1	GATCATTTATCTTTCACTGCGGAGAAGTTT	GMP-gRNA-P-1
	gRNA-Pri-2	gcagtgaaagataaatgatcTTTTTGAGTGTTGGGACCCTgttttagagctagaaa	
GUA1	gRNA-Pri-1	GATCATTTATCTTTCACTGCGGAGAAGTTT	GMP-gRNA-P-2
	gRNA-Pri-3	gcagtgaaagataaatgatcTTTAACTTCCCTGGGTCATTgtttagagctagaaa	
ZWF1	gRNA-Pri-1	GATCATTTATCTTTCACTGCGGAGAAGTTT	GMP-gRNA-P-3
	gRNA-Pri-4	gcagtgaaagataaatgatcCCCGGTTTCGGCTCGGCCGGgttttagagctagaaa	
RKI1	gRNA-Pri-1	GATCATTTATCTTTCACTGCGGAGAAGTTT	GMP-gRNA-P-4
	gRNA-Pri-5	gcagtgaaagataaatgatcGCAATTTATTCTCTTTGTACgttttagagctagaaa	
STB5	gRNA-Pri-1	GATCATTTATCTTTCACTGCGGAGAAGTTT	GMP-gRNA-P-5
	gRNA-Pri-6	gcagtgaaagataaatgatcGGTATAATATCCGAGCGTACgtttagagctagaaa	
RAP1	gRNA-Pri-1	GATCATTTATCTTTCACTGCGGAGAAGTTT	GMP-gRNA-P-6
	gRNA-Pri-7	gcagtgaaagataaatgatcAACGAAGAAGAGGCGGGTAGgttttagagctagaaa	

Table S2. Primer sequences for construction of integration cassettes to overexpress target genes.

Target gene	Primer name	Primer sequence	Template DNA
IMD3	Integ-Pri-1	ATTACATAAATATTAGCCTTCAAAGCCGCACACGTTTCTTTTTCCGAAAAAGTTTATCATTATCAATACTCGCCATTTCAAAGAATACGT	pRS416-GPDp
	Integ-Pri-2	AAGCTCTTGGCGAATTCCAAGGCAGTCTTGTAGTCTCTAACGGCGGCCATATCCGTCGAAACTAAGTTCTGGTGTTTTA	
GUA1	Integ-Pri-3	TTAGTTGAGTTATAGTTTCGGTCACGGTAGTCTCTAATTAGCTTGCGTTCAGTTTATCATTATCAATACTCGCCATTTCAAAGAATACGT	pRS416-GPDp
	Integ-Pri-4	TAAGATAGTGTCAAACATGTTAGAAACTTGTTCACCGGCAGCCATATCCGTCGAAACTAAGTTCTGGTGTTTTA	
ZWF1	Integ-Pri-5	AAGAACAACAATAATAGTAGCGCTACTGGAAGCACCACGTAATAGTGGAAAGTTTATCATTATCAATACTCGCCATTTCAAAGAATACGT	pRS416-GPDp
	Integ-Pri-6	AAGACAGATATGACGGTATTTTTTTCGAATTTGACGGGGCCTTCACTCATATCCGTCGAAACTAAGTTCTGGTGTTTTA	
RKI1	Integ-Pri-7	GCCCAGATTGCTTGTGGCCGAGGCAGTGAAAAGTCGCGATGAGTTGAAAATTCGCGGCCACCTACGCCGCTATCTTTGCAACAACTATCT	pRS416-CCW12p
	Integ-Pri-8	GCCCAAAGATTCTAACGCATCAATTTTTGGGACACCGGCAGCCATTATTGATATAGTGTTTAAGCGAATGACAGAAGATTA	
STB5	Integ-Pri-9	GGAATATAACGACAAGGCCGATACCACGGGAAAAATAGGGCGAGCGGAAACGATTTGGGCGCGAATCCTTTATTTTGGCTTCACCCT	pRS426-PGK1p
	Integ-Pri-10	GTAGTACGTTGTGATCTCCCGCCTTGATGTGCAAAATTGGGACCATCCATTGTTTTATATTTGTTGTAAAAAGTAGATAATTACTTCCTT	
RAP1	Integ-Pri-11	ACGGGCACGATGCATCCATGGGACTAAACCTACGGAGACAGACCAAAGGTTTCGCGGCCACCTACGCCGCTATCTTTGCAACAACTATCT	pRS416-CCW12p
	Integ-Pri-12	GCATCAACATATTCTGCTGGTGCAGTTTCAAAATCATCTGGACTAGACATTATTGATATAGTGTTTAAGCGAATGACAGAAGATTA	

Table S3. Primer sequences for confirmation of cassette integration.

Target gene	Primer name	Primer sequence
IMD3	Confirm-Pri-IMD3-F	GCTAGGATATCGGGAAAAAAGAGACAAGGGAACTTGAAAAAC
	Confirm-Pri-IMD3-B	GACCTGGCAAAACCAAAAAGTCGTTATAAGTCAACCCACCTCT GGCTAGAAA
GUA1	Confirm-Pri-GUA1-F	ATATGCTTTCATCCTCTCGCTCCCCTACAATGGCTTC
	Confirm-Pri-GUA1-B	CTTAATCTTCTGGTAATCAAATGAGAGTATTGAGA
ZWF1	Confirm-Pri-ZWF1-F	AAAGGATCTCGTCTCTGTTGGGAGCACCTGGTAAGTAAG
	Confirm-Pri-ZWF1-B	CCATGGACAATTTGGACCGGGCATAACCGAAGATCTTG
RKI1	Confirm-Pri-RKI1-F	CATCCGGTAAGTCCGCCACACTATA
	Confirm-Pri-RKI1-B	GCTACCACTACCAATTCCAATAATT
STB5	Confirm-Pri-STB5-F	ACTCCATTGTTGATGCAAAAGTTGTA
	Confirm-Pri-STB5-B	CCACACTTCTTCTTTAATTTTCTGC
RAP1	Confirm-Pri-RAP1-F	AAGAACTGGTCAAAAACATCACGTGAAAAG
	Confirm-Pri-RAP1-B	GTTTTCTTCGTTTTGATTAGCTTTTACCTC

Table S4. Primer sequences for comparison of mRNA expression level.

Target gene	Primer name	Primer sequence
ACT1	qPCR-ACT1-F	ATTATATGTTTAGAGGTTGCTGCTTTGG
	qPCR-ACT1-B	CAATTCGTTGTAGAAGGTATGATGCC
IMD3	qPCR-IMD3-F	GCCAGGTCTGGTTGATTTCC
	qPCR-IMD3-B	CCACCCAACAAAGCCATGAA
GUA1	qPCR-GUA1-F	TTGGTCGTGGTGACAAGAGA
	qPCR-GUA1-B	TTATCGGAGGTGGCAATGGT
ZWF1	qPCR-ZWF1-F	GTGGCCAAGCAGATCAAGAG
	qPCR-ZWF1-B	GGCCGAAAGGTTTCTCTACG
RKI1	qPCR-RKI1-F	TCCTTTGGAGGATGCCAAGA
	qPCR-RKI1-B	ACCACTGTGCTACCACTACC
STB5	qPCR-STB5-F	ATTCGATTCTGTGCCGGTTG
	qPCR-STB5-B	GTATGCGGCAATGAACCTGT
RAP1	qPCR-RAP1-F	TAAGCAGCACCGCATCAATC
	qPCR-RAP1-B	TGTCTTCGTCGTTGACGTTG
